# Systematic study of a corrugated waveguide as a microwave undulator

**DOI:** 10.1107/S1600577518014297

**Published:** 2019-01-01

**Authors:** Liang Zhang, Wenlong He, Jim Clarke, Kevin Ronald, Alan D. R. Phelps, Adrian Cross

**Affiliations:** a Department of Physics, SUPA, University of Strathclyde, Glasgow G4 0NG, UK; b The Cockcroft Institute, Sci-Tech Daresbury, Keckwick Lane, Daresbury, Warrington WA4 4AD, UK; c ASTeC, STFC Daresbury Laboratory, Sci-Tech Daresbury, Keckwick Lane, Daresbury, Warrington WA4 4AD, UK

**Keywords:** microwave undulator, free-electron laser, corrugated waveguide, balanced hybrid condition

## Abstract

Microwave undulators have great potential to be used in short-wavelength free-electron lasers. In this paper, the properties of a corrugated waveguide and its performance as an undulator cavity for a UK X-ray free-electron laser were systematically studied. The equations presented in this paper allow a fast estimation of the dimensions of the corrugated waveguide. An undulator cavity operating at 36 GHz designed for the HE_11_ and HE_12_ modes was investigated and the performance of both modes compared.

## Abstract 2

A microwave undulator operating at 36 GHz has been designed for a UK X-ray free-electron laser. The equivalent magnetic field is 1.27 T when driven by 50 MW of input power.

## 1. Introduction   

Undulators are one of the most important components in a free-electron laser (FEL) (Deacon *et al.*, 1977 ▸; Huang & Kim, 2007 ▸). Currently, the conventional permanent magnet undulator (PMU) plays the dominant role. Microwave undulators (MUs) (Shintake *et al.*, 1982 ▸, 1983 ▸) that also have a periodic magnetic field can be potential undulators and have the following advantages. (i) Fast dynamic control of the polarization (Shumail & Tantawi, 2016 ▸). (ii) Easy control of the field strength, which can be adjusted through the input microwave power, whereas, in a PMU, mechanical methods to adjust the magnet gap or the magnet period have to be used which can be complicated. (iii) It is challenging for a PMU to achieve short periods as the magnetic field strength would be significantly reduced. In contrast, for an MU the equivalent period is mainly determined by the wavelength of the electromagnetic wave, therefore a short period can be achieved if the MU operates at a higher frequency. (iv) The MU is essentially a metallic cavity and hence it is robust against damage by ionizing radiation near electron beam dump regions, as compared with the PMUs that are made of rare-earth materials, which may be more susceptible to damage in harsh ionizing radiation environments.

However, since the concept of the microwave undulator was proposed in 1982 (Shintake *et al.*, 1982 ▸), progress has taken longer than expected, mainly due to the limited availability of high-power microwave sources. In 1983 (Shintake *et al.*, 1983 ▸), the first MU experiment was carried out and an equivalent magnetic field *B*_u_ of 0.045 T with an undulator parameter *K* of 0.24 was achieved when driven by a 300 kW, 2.856 GHz microwave source. A ridged rectangular cavity was used and a quality factor *Q* of 7100 was measured. To achieve a similar performance to a state-of-the-art PMU, for example, 1.29 T for a 15 mm-period PMU used in the Swiss-FEL at the Paul Scherrer Institute, the required driving power would need to be more than 20 MW at 10.5 GHz (assuming the same *Q* factor of 7100 can be achieved for a similar structure scaled for operation at 10.5 GHz). However, the electric field at the wall of the ridged rectangular cavity would be too high and susceptible to microwave breakdown. A significant improvement on the MU was made with the use of a low-loss HE_11_ mode in a corrugated waveguide. A cavity made of a corrugated waveguide operating at the X-band was measured and it was able to achieve a *Q *factor as high as 91 000. When it was driven by a 50 MW SLAC klystron at 11.424 GHz (Tantawi *et al.*, 2014 ▸), such an MU was able to achieve an equivalent *B*_u_ of 0.65 T with a period of 13.9 mm.

The corrugated waveguide is normally overmoded. The numerical calculation of the eigenmodes of the cavity made with the corrugated waveguide is time-consuming. In this paper, analytical equations and design constraints are summarized which could be used to quickly estimate the dimensions of the structure. The microwave undulator can be operated at a high millimetre-wave frequency to achieve a smaller period. In this paper, a corrugated waveguide operating at 36 GHz was designed as an MU for a UK XFEL.

The paper is organized as follows. In Section 2, the principle of the MU and the relation between the cavity and the microwave sources is presented. Section 3 introduces the theory of the corrugated waveguide and the equations that determine the dimensions, field distribution and the loss coefficient. Section 4 details the simulation results of an MU operating at 36 GHz. Both the HE_11_ and HE_12_ operating modes were investigated and their performances compared.

## 2. Principles of MU   

The principle of the MU can be found in the literature (Pellegrini, 2006 ▸; Seidel, 2001 ▸; Shintake *et al.*, 1983 ▸; Shumail & Tantawi, 2016 ▸). The relativistic electrons in an MU cavity will interact with both the electric field *E_x_* = *E*_0_ sin(2π*z*/λ_g_) sin(ω*t*) and magnetic field *B_y_* = *B*_0_ cos(2π*z*/λ_g_) cos(ω*t*). Compared with a PMU, the Lorentz force in an MU can be rewritten in the form
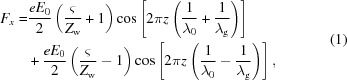
where *e* is the charge on the electron, λ_0_ is the free space wavelength, and λ_g_ is the wavelength of the electromagnetic wave in the undulator cavity. *E*_0_ and *B*_0_ are the peak electric and magnetic field strength in the microwave undulator cavity, respectively. *Z*_w_ is the wave impedance in the cavity and ς is the wave impedance in free space. The second term leads to a long wavelength and is undesirable in the undulator of a short-wavelength FEL. The second term can be ignored if the wave impedance is close to the free-space impedance, which means the operating frequency is far from the cut-off frequency of the waveguide. In this case, the equivalent magnetic field *B*_u_ and wavelength λ_u_ of the microwave undulator are given by
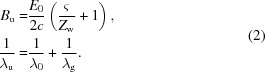
If the microwave source starts to fill the cavity at time zero, the stored energy at time *t* in the cavity can be expressed as (Alvarez, 1986 ▸)
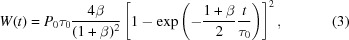
where *P*_0_ is the input power, τ_0_ = *Q*_0_/ω, and β = *Q*_0_/*Q*_e_. *Q*_0_ and *Q*_e_ represent the intrinsic and external quality factor of the cavity. In a steady state, where *t* ≫ τ_0_, the stored energy reaches its maximum value of *P*_0_τ_0_ if the coupling aperture is specifically designed to achieve β = 1. The input power in this case will be equal to the Ohmic loss in the cavity. In the cases of an under-coupled (β < 1) and over-coupled (β > 1) regime, the stored energy in the steady state is smaller. The filling time of the cavity *t*_tf_ can be calculated if a charging factor η, defined as the ratio of the charged energy and its maximum value, is known,
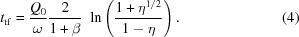
From equations (3) and (4), to achieve maximum storage at a given input power, a higher *Q* factor of the cavity is preferred; however, the filling time increases simultaneously. In applications of MUs, high-power microwave sources with output powers of megawatts or more are needed to achieve a high equivalent magnetic field. Such high-power microwave sources normally operate in pulsed mode to reduce the power supply requirements and associated thermal stress. The filling time of the MU cavity should match the pulse length of the microwave source. The coupler can be slightly over-coupled to reduce the filling time while maintaining a high *Q* factor. If the input microwave frequency is 36 GHz, with a 2 µs pulse length, the *Q* factor of the cavity should be under 150 000 if β = 1 and η = 0.9.

The electric field strength inside the cavity along the electron beam path can be calculated by careful selection of the operating mode of the cavity. Knowing the input power and *Q* factor, the parameters of the MU can be determined.

## 3. Corrugated waveguide   

**Figure 1:**
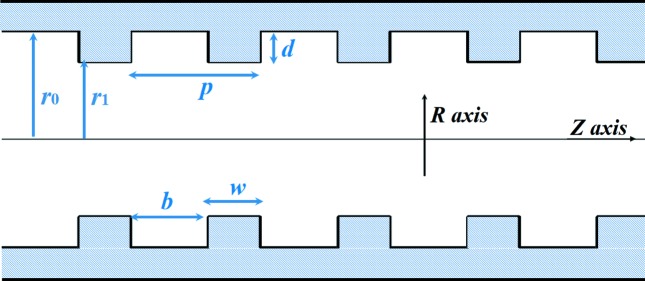
Schematic drawing of the corrugated waveguide.

A high-*Q* low-loss cavity is of great importance when it is used as an MU. In a circular waveguide, the mode with the lowest loss is the TE_01_ mode. However, the field strength at the waveguide center for this mode is small and hence is not a good option for an MU as the electrons are to propagate down the center of the structure. A corrugated waveguide, as shown in Fig. 1 ▸, can be used in other applications such as a feed horn for a transmission line system due to its advantages of low cross-polarization field, low loss and wide bandwidth. Its low attenuation feature is attractive for the transmission of high-power microwaves. The fundamental mode HE_11_ was found to have lower loss compared with the TE_01_ mode in the circular waveguide. It has been used to transport megawatt-level millimetre-wavelength radiation generated by gyrotron oscillators for fusion experiments such as ITER. In recent years, the corrugated waveguide has been proposed as an MU (Shumail *et al.*, 2011 ▸; Chang *et al.*, 2012 ▸; Toufexis & Tantawi, 2017 ▸).

The corrugated waveguide contains circular waveguide steps and smooth sections. The properties of a corrugated waveguide with arbitrary radial corrugation depth can be accurately solved using a mode-matching method (James, 1981 ▸; Neilson *et al.*, 1989 ▸; Zhang *et al.*, 2016 ▸). For the periodically corrugated waveguide used for transmitting the microwave power, analytical equations can be derived based on the fact that the waveguide radius is larger than the wavelength. The propagation characteristics of the periodically corrugated waveguide were studied using the simplified surface-impedance approach or the rigid equations taking into account the spatial harmonics in the corrugation gaps (Clarricoats & Olver, 1984 ▸; Dragone, 1980 ▸; Clarricoats & Saha, 1971 ▸; Kowalski *et al.*, 2010 ▸).

The surface-impedance approach assumes that only the lowest transverse magnetic (TM) standing wave exists in the slot and ignores its spatial harmonics. It gives a good approximation when the period per wavelength, defined by λ_0_/*p*, is a reasonably large value and the corrugation slot length, defined by *w* = *p* − *b* in Fig. 1 ▸, is a small value. At low frequency, for example in the application of the microwave undulator, these assumptions would normally be satisfied. If only the lowest TM standing wave were present in the corrugation slot, its surface admittance at *r* = *r*_1_ can be written as
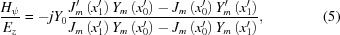
where *m* indicates the azimuthal mode number, *x*_1_′ = *kr*_1_ and *x*_0_′ = *kr*_0_. *J_m_* and *Y_m_* are the first and second kind of Bessel functions of order *m*, respectively. *Y*_0_ is the free-space wave admittance. The surface admittance becomes 0 when
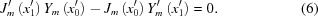
This is known as the balanced hybrid condition. If the operating frequency (*f*) and the waveguide radius (either *r*_0_ or *r*_1_) are given, the corrugation depth *d* = *r*_0_ − *r*_1_ can be determined from the equation. At *m* = 1 and *x*_1_′ ≫ 1, the surface admittance can be further simplified as 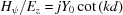. The corrugation depth *d* would be equal to λ_0_/4 to ensure *H*_ψ_ = 0.

Under the balanced hybrid condition and *x*_1_′ ≫ 1, the dispersion curve between *k* and *k_z_* is determined by
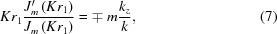
where *K* ^2^ = *k* ^2^ − *k*_*z*_^2^, the ‘−’ in ‘
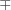
’ denotes the HE modes, and the ‘+’ denotes the EH modes. For a large radius, which leads to *k_z_* ≃ *k* and *m* = 1, equation (7) can be further simplified to *J*_0_(*Kr*_1_) = 0 for HE_1n_ modes and *J*_2_(*Kr*_1_) = 0 for EH_1*n*_ modes (Shumail, 2014 ▸).

**Figure 2:**
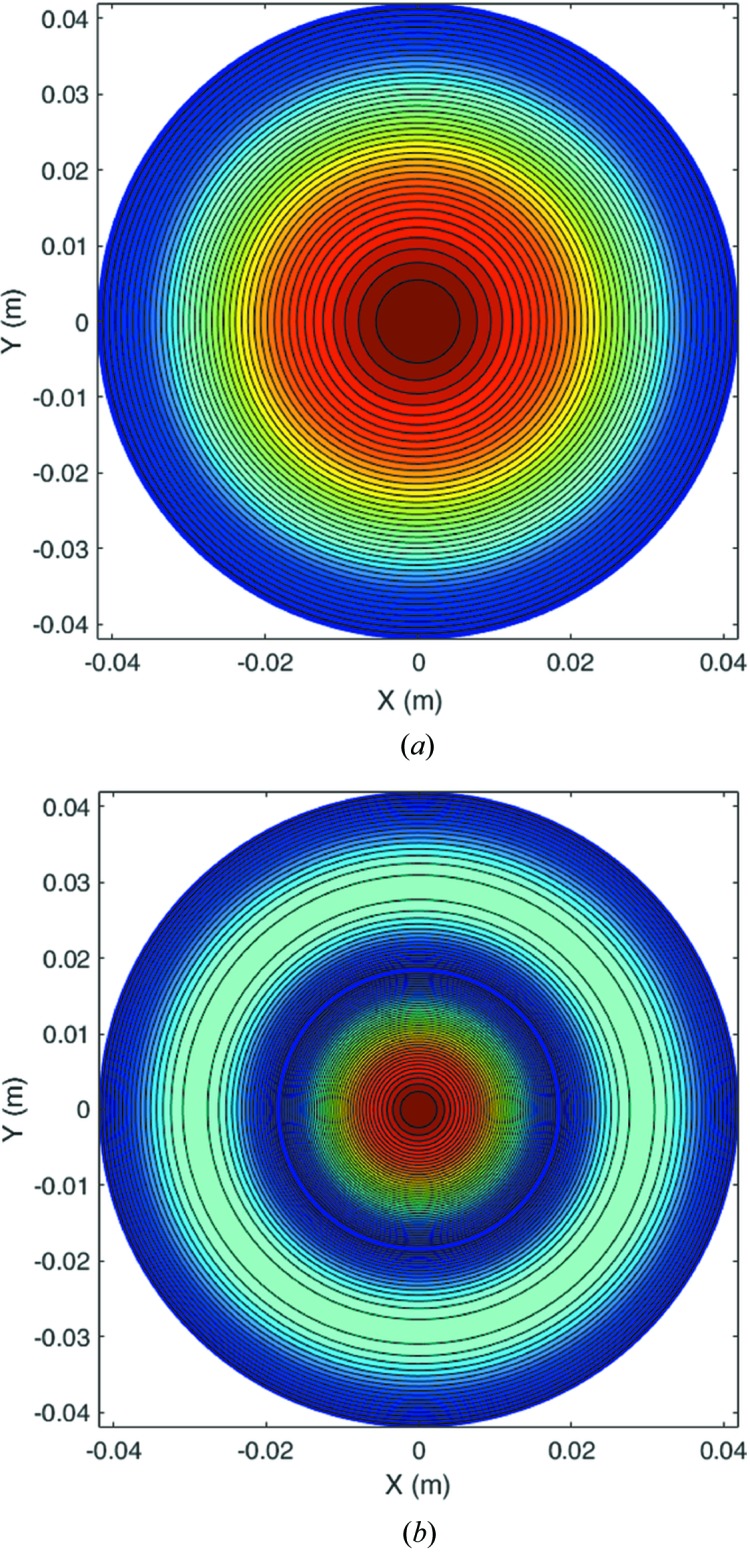
Contour plot of the electric field patterns of the (*a*) HE_11_ and (*b*) HE_12_ modes.

To describe the field distribution inside the corrugated waveguide, different eigenmode sets using TE/TM and HE/EH combinations have been derived (Crenn & Charollais, 1996 ▸). The field inside the corrugated waveguide with the linearly polarized mode sets can be simplified as (Clarricoats & Saha, 1971 ▸)
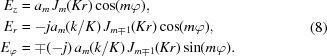
Usually, a higher order HE or EH mode is not linearly polarized. The suitable operating modes in the corrugated waveguide for the microwave undulator application are the HE_11_ and HE_12_ modes because they are low loss, linearly polarized and have the peak electric field at the waveguide center. The electric field patterns of the HE_11_ and HE_12_ modes are shown in Fig. 2 ▸. The HE_12_ mode has larger field density at the waveguide center. It can have a bigger electric field compared with the HE_11_ mode at the same input power.

The attenuation coefficient defined by the ratio between the lost power and the transported power per meter for the HE_1*n*_ modes, under the balanced hybrid condition, can be written as (Clarricoats & Olver, 1984 ▸; Doane, 1985 ▸)
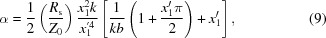
where *Z*_0_ = 1/*Y*_0_ is the free space wave impedance, *R*_s_ = (π*f*μ_0_σ)^1/2^ is the resistivity of the corrugated metal waveguide and σ is the conductivity of the metal. In this paper, oxygen-free high-conductivity copper was chosen and σ = 5.8 × 10^7^ S m^−1^ was used in the simulation. Equation (9) implies that the attenuation coefficient is proportional to *r*_1_^−3^ and *f* ^−2^ in the case of *x*_1_′ ≫ 1.

The corrugated waveguide can be shortened at both ends to form a cavity. The resonance frequency of the corrugated waveguide can be estimated as
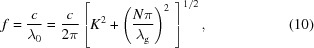
where 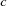 is the speed of light, *N* is an integer and λ_g_ is the wavelength of the resonance mode in the cavity. Because the corrugated waveguide is normally overmoded, λ_0_ ≃ λ_g_ when *N* = 2. As mentioned earlier, the surface-impedance approach gives a good approximation when λ_0_/*p* is a reasonably large value. Therefore, when designing the corrugated waveguide, the period *p* can be selected to be smaller than λ_0_/2. It is of course the case that ‘the smaller the period, the better’; however, as the operating frequency increases, the wavelength becomes smaller, resulting in a small corrugation period and a thin corrugation slot of *w* ≃ 0 which significantly increases the machining difficulty. The final choice of the geometry should therefore also consider the machining tolerance.

Since 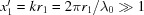 has to be satisfied, the corrugated waveguide radius *r*_1_ is a large value. As the attenuation coefficient is proportional to *r*_1_^−3^, it is preferable to have a large 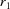 at a given operating frequency. From equation (8), the waveguide radius affects the field strength if the input power is a fixed value. In a microwave undulator, a high field at the electron beam path, in this case the waveguide center, is desired. Therefore 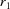 should be as small as possible under the constraint of *kr*_1_ ≫ 1. On the other hand, it is preferred for the electron beam that travels through the microwave undulator to see a uniform field in the radial direction. The minimum waveguide radius can be solved from the field pattern of the operating mode if the electron beam aperture *R*_b_ and a threshold, for example 90% of the maximum field at the beam edge, are defined. For HE_1*n*_ modes, it follows that *J*_1_(*KR*_b_) = 0.9. Taking the first two solutions of *J*_0_(*Kr*_1_) = 0, *K* is approximately equal to 2.4/*r*_1_ or 5.5/*r*_1_ for the HE_11_ or the HE_12_ mode, respectively. Therefore the following parameters were chosen,
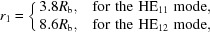
resulting in a reasonable value of *r*_1_. Meanwhile the value of *r*_1_ should satisfy *x*_1_′ = 2π*r*_1_/λ_0_ ≫ 1. If *x*_1_′ = 5 is used, then *r*_1_ ≥ 0.8λ_0_ and the results become
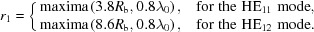



## 4. Design of a corrugated waveguide for an MU   

A corrugated waveguide cavity operating at 36 GHz was designed as a potential microwave undulator for the UK XFEL. The initial geometry parameters were calculated from the equations described in previous sections. Both of the HE_11_ and HE_12_ modes are considered. A summary of the geometry parameters as well as the undulator deflection parameter 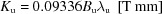 are listed in Table 1 ▸.

**Figure 3:**
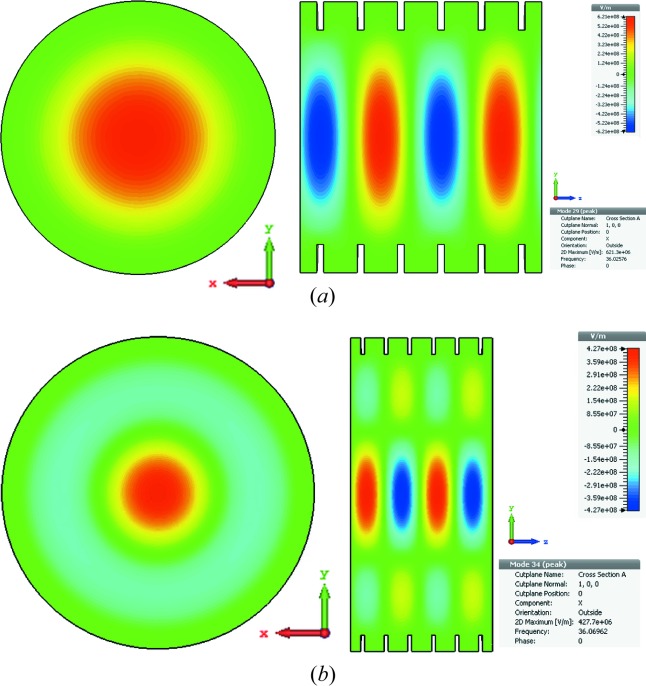
Electric field patterns of the (*a*) HE_11_ and (*b*) HE_12_ modes.

The accurate resonance frequency and *Q* factor of the designed structure were simulated using CST Microwave Studio. The simulated structure was set at six corrugation periods to significantly reduce the simulation time. For such a short cavity length, the loss at the conducting wall at both sides will be much larger than the loss at the corrugated wall because the field strength at the corrugated wall is much smaller. In the simulation, a periodic boundary condition was set at both ends of the corrugated waveguide. In this way, the loss at both sides will not be included. Fig. 3 ▸ shows the electric field distribution of the cavity modes of HE_114_ and HE_124_. The peak *Ex* field along the *z* axis was obtained from the field distribution of the eigenmode and normalized to an Ohmic loss power of 50 MW at a cavity length of 1 m. With the same input power and similar operating frequency, the waveguide radius of the HE_12_ mode was about 2.2 times that of the HE_11_ mode, and its *Q* factor was nearly 2 times that of the HE_11_ mode. The *Ex* field strength was slightly smaller than that of the HE_11_ mode. The equivalent magnetic field *B*_u_ was also similar for both cases.

**Figure 4:**
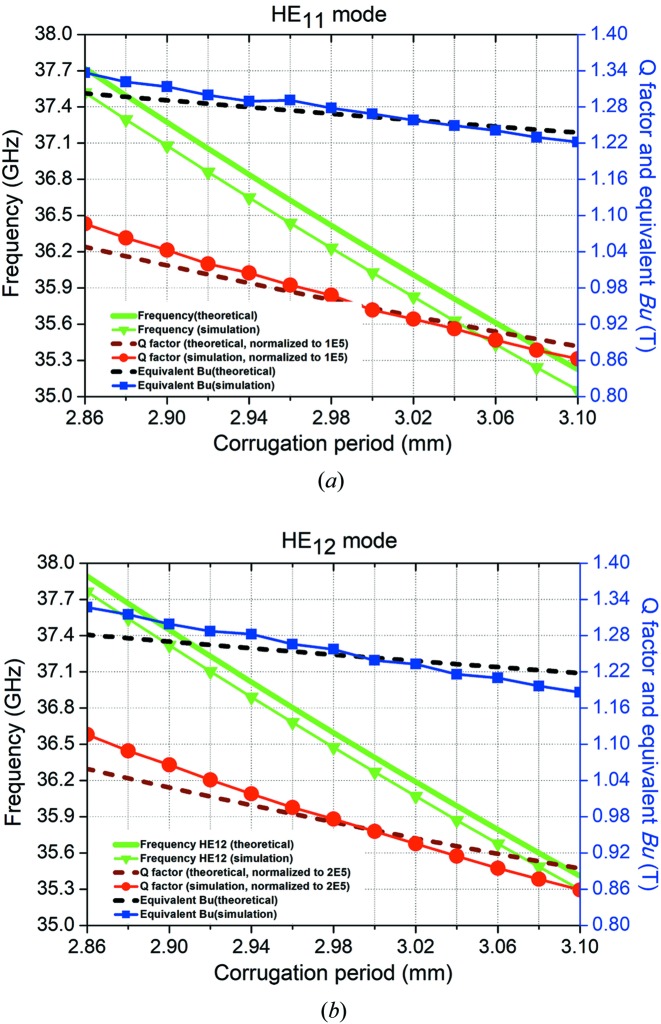
Simulation results of different corrugation periods: (*a*) HE_11_ mode and (*b*) HE_12_ mode.

Parameter scans were used to study the effects of varying of the corrugation geometry parameters such as the corrugation period (*p*), the slot length (*w*) and the waveguide radius (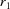). The results of resonance frequency, *Q* factor and equivalent magnetic field strength are shown in Figs. 4 ▸–6. The HE_11_ mode has a similar trend to the HE_12_ mode. In Fig. 4 ▸, as the corrugation period increases, the resonance frequency becomes smaller while the slot length (*w*) and the waveguide radius (*r*_1_) are kept the same. The results matched the theoretical calculation well from equation (10) and the scaling law equations (Shumail *et al.*, 2011 ▸). The difference between the simulation and theoretical calculation is less than 2%, therefore it allows a fast prediction of the cavity parameters from the theoretical analysis. The *Q* factor reduces as the corrugation period increases. It is mainly caused by the increase of the overall waveguide length and the decreases in the stored energy per unit length.

**Figure 5:**
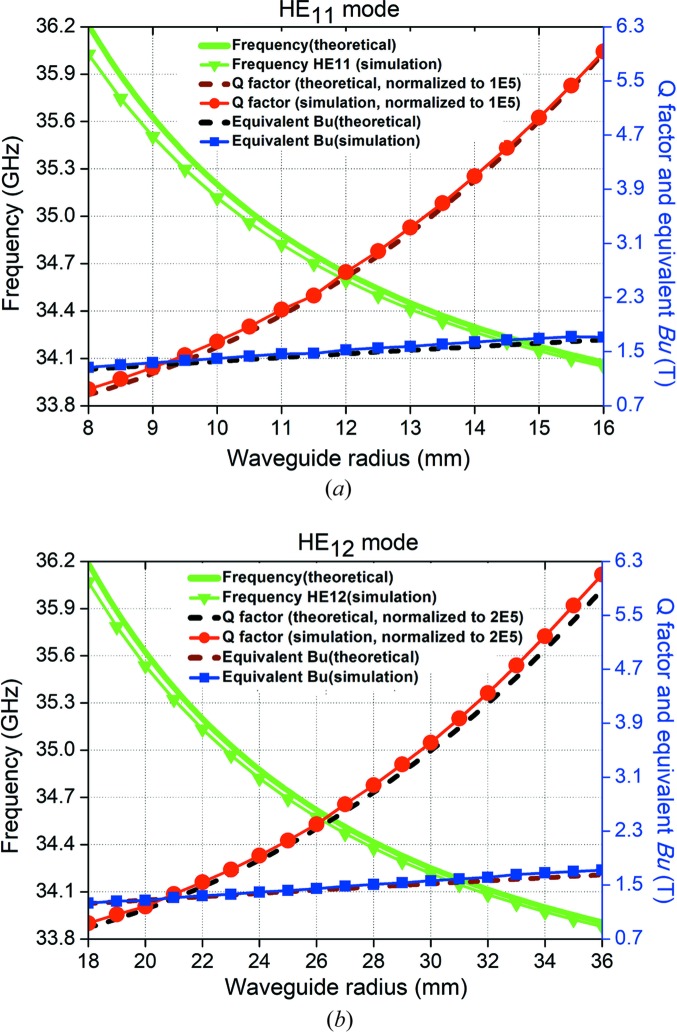
Simulation results of different waveguide radii: (*a*) HE_11_ mode and (*b*) HE_12_ mode.

The simulation results at the different waveguide radii are shown in Fig. 5 ▸. The trends for the HE_11_ and HE_12_ modes are very similar. Increasing the waveguide radius would significantly increase the *Q* factor and the resonance frequency drops quickly. However, from equation (4), a high *Q* factor would result in a long filling time. For the HE_11_ mode, a 20% increment of the waveguide radius would lead to a *Q* factor close to 150 000, requiring an increase in the pulse length of the high-power microwave source used to drive the structure. Also, increasing the waveguide radius did not improve the equivalent magnetic field, which was almost the same. Therefore, the designed waveguide radius was considered to be optimal.

**Figure 6:**
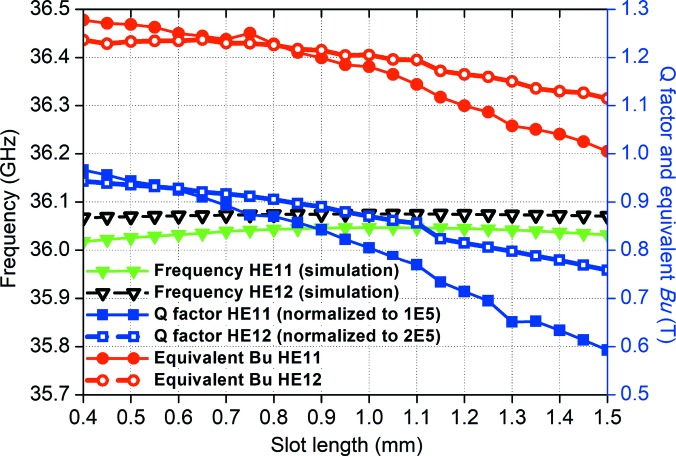
Simulation results of different slot lengths.

The simulation results of the variation in slot length while keeping the corrugation period constant are shown in Fig. 6 ▸. Only the CST simulation results were included as the theoretical analysis will not be accurate at small slot length values. As the slot length increases, the assumption of only the lowest TM standing wave appearing in the slot is no longer satisfied. The field at the corrugation wall became larger and caused a significant reduction in the cavity *Q*. The *Q* factor dropped more quickly for the HE_11_ mode than the HE_12_ mode because its waveguide radius was smaller. As the corrugation period was kept constant, the change in the resonance frequency was small. The equivalent magnetic field *B*_u_ also changed by a small amount. The simulation results proved that the slot length should be as small as possible to achieve a high *Q* factor. However, the difficulty in manufacturing such a structure while maintaining sufficient mechanical strength needs to be taken into account. A slot length of 0.5 mm is a reasonable value to satisfy the criteria of ease of manufacture, while at the same time being mechanically strong.

## 5. Discussion and conclusions   

In this paper, a corrugated waveguide was studied as a microwave undulator for a UK XFEL. The equations that govern the performance of the microwave undulator are presented and have been used to estimate the dimensions of the corrugated waveguide.

Both of the HE_11_ and HE_12_ modes existing in the corrugated waveguide are suitable operating modes. Their characteristics were studied using numerical simulation and their performance compared in the design of a corrugated waveguide operating at 36 GHz. It was found that the equivalent magnetic field for both of the modes was very similar. The HE_12_ mode had a much larger waveguide radius, which could be advantageous for high-frequency operation because it helps to reduce the difficulty in manufacture as well as reducing the electric field at the wall. The HE_12_ mode is also less sensitive to the slot length compared with the HE_11_ mode. The drawback of the HE_12_ mode includes a much higher *Q* factor that leads to a longer filling time and greater sensitivity to the waveguide period.

## Figures and Tables

**Table 1:** Parameters of the MU composed of a corrugated waveguide

Operating mode	HE_11_	HE_12_
Operating frequency (GHz)	36	36
λ_0_ (mm)	8.33	8.33
*R*_b_ (mm)	2.0	2.0
*r*_1_ (mm)	4*R*_b_ = 8.0	9*R*_b_ = 18.0
*d* = λ_0_/4 (mm)	2.1	2.1
λ_g_ (mm)	9.06	9.12
*p* = λ_g_/3 (mm)	3.00	3.02
*w* (mm)	0.5	0.5
*b* = *p* − *w* (mm)	2.50	2.52
*Q* factor	94344	187073
Input power (MW)	50	50
Peak *Ex* on axis (V m^−1^)	3.8 × 10^8^	3.7 × 10^8^
*B*_u_ (T)	1.27	1.23
λ_u_ (mm)	4.34	4.35
*K*_u_	0.52	0.50
